# Relative, not absolute, stimulus size is responsible for a correspondence effect between physical stimulus size and left/right responses

**DOI:** 10.3758/s13414-022-02490-7

**Published:** 2022-04-22

**Authors:** Peter Wühr, Melanie Richter

**Affiliations:** Institut für Psychologie, Technische Universität Dortmund/TU Dortmund University, Emil-Figge-Straße 50, 44227 Dortmund, Germany

**Keywords:** Compatibility effect, Correspondence effect, Physical stimulus size, Response location, Relative, Absolute, Context-dependent, Context-independent

## Abstract

Recent studies have demonstrated a novel compatibility (or correspondence) effect between physical stimulus size and horizontally aligned responses: Left-hand responses are shorter and more accurate to a small stimulus, compared to a large stimulus, whereas the opposite is true for right-hand responses. The present study investigated whether relative or absolute size is responsible for the effect. If relative size was important, a particular stimulus would elicit faster left-hand responses if the other stimuli in the set were larger, but the same stimulus would elicit a faster right-hand response if the other stimuli in the set were smaller. In terms of two-visual-systems theory, our study explores whether “vision for perception” (i.e., the ventral system) or “vision for action” (i.e., the dorsal system) dominates the processing of stimulus size in our task. In two experiments, participants performed a discrimination task in which they responded to stimulus color (Experiment 1) or to stimulus shape (Experiment 2) with their left/right hand. Stimulus size varied as an irrelevant stimulus feature, thus leading to corresponding (small-left; large-right) and non-corresponding (small-right; large-left) conditions. Moreover, a set of smaller stimuli and a set of larger stimuli, with both sets sharing an intermediately sized stimulus, were used in different conditions. The consistently significant two-way interaction between stimulus size and response location demonstrated the presence of the correspondence effect. The three-way interaction between stimulus size, response location, and stimulus set, however, was never significant. The results suggest that participants are inadvertently classifying stimuli according to relative size in a context-specific manner.

## Introduction

The term *stimulus-response compatibility* (S-R compatibility; SRC) refers to the fact that some mappings between a particular set of stimuli and a particular set of responses allow for better performance than other mappings (Alluisi & Warm, 1990; Proctor & Vu, 2006). A mapping that improves performance in terms of speed and/or accuracy is called a “compatible” mapping or condition, whereas a mapping that impairs performance is called an “incompatible” mapping or condition. Investigating S-R compatibility is valuable both for basic and for applied research. In basic research, investigating S-R compatibility yields insights into the mental representations of stimuli and responses, and about the processes that “translate” stimuli into responses (Alluisi & Warm, 1990; Kornblum et al., 1990; Proctor & Vu, 2006). In applied research, investigating S-R compatibility yields important guidelines for designing safe environments, devices, and tasks (Proctor & Vu, 2010; Wickens et al., 2003).

According to Kornblum et al. (1990), S-R compatibility arises from “dimensional overlap” between a stimulus set and a response set. In particular, dimensional overlap describes a relationship (i.e., similarity) between stimulus and response sets on a perceptual, conceptual, or structural level (cf. Kornblum et al., 1990; Kornblum & Lee, 1995). The well-known spatial compatibility effect (e.g., Fitts & Seeger, 1953) rests on dimensional overlap on a *perceptual* level. Here, stimuli and responses vary on the same spatial dimension, and you can literally “see” the match or mismatch between stimuli and responses. In a typical task, both stimuli and responses can occur on a left or a right location. In the compatible condition, participants have to make a response that matches the relative position of the stimulus (e.g., left-left, right-right). In the incompatible condition, participants have to make a response that does not match the relative position of the stimulus (e.g., left-right, right-left). Performance in compatible conditions is much better than performance in incompatible conditions (e.g., Fitts & Seeger, 1953; see Proctor & Vu, 2006, for a review). A similar phenomenon – called the “Simon” effect – occurs when stimulus location is irrelevant for the task, and participants respond to a nonspatial stimulus feature (Simon & Rudell, 1967; see Hommel, 2011, for a review).^1^

Dimensional overlap on a conceptual level provides the basis for semantic compatibility effects. A typical task might include the stimulus words “left” and “right” and keypress responses on a left or right location. In compatible conditions, stimuli and responses are referring to the same value on the shared conceptual dimension, whereas this is not the case in incompatible conditions. As a result, compatible conditions again allow for better performance than do incompatible conditions (e.g., Wang & Proctor, 1996).

Whereas dimensional overlap on perceptual or conceptual levels is readily seen or recognized by most people, dimensional overlap on a structural level is not. In fact, structural relationships between stimulus and response sets may be subtle, and sometimes surprising. A prominent example for a compatibility effect that rests on structural overlap is the spatial numerical associations of response codes (SNARC) effect (Dehaene et al., 1990, 1993; Fias & Fischer, 2005). In its original formulation, the SNARC effect refers to the observation that left responses are faster and more accurate than right responses to small numbers, whereas the opposite is true for larger numbers. Many authors assume that numbers have an analog spatial representation in the cognitive system, called the “mental number line,” on which numbers are ordered from left to right, and thus share the same left-to-right structure in space as left and right response locations (e.g., Dehaene et al., 1993; Kornblum & Lee, 1995).

Further studies investigated whether absolute or relative numerical size is crucial for the SNARC effect. In their seminal study, Dehaene et al. (1993) compared SNARC effects for a stimulus set ranging from 0 to 5 to SNARC effects for a stimulus set ranging from 4 to 9. While the numbers 4 and 5 were associated with the right response in the former interval, in which they were relatively large, the same numbers were associated with the left response in the latter interval, in which they were relatively small. Hence, these results showed that relative but not absolute numerical size interacts with response location in producing the SNARC effect (see Ben Nathan et al., 2009, and Fias et al., 1996, in replicating and extending these findings).

To explain differences in the processing of compatible and incompatible conditions, authors proposed so-called “dual-route models” (e.g., Barber & O’Leary, 1997; De Jong et al., 1994; Kornblum et al., 1990). These models assume two separate pathways, or routes, through which a stimulus can activate a response. Through the “indirect” route, controlled processes translate the relevant stimulus into the correct response according to the instructions (S-R rules) stored in working memory. This route is assumed to rest on short-term associations, which can be established on the fly in working memory (e.g., Tagliabue et al., 2000). In contrast, through the “direct” route, both relevant and irrelevant stimulus features can activate a compatible or corresponding response in an automatic fashion. The direct route is assumed to rest on long-term associations, which may represent dimensional overlap between stimulus and response dimensions (e.g., Tagliabue et al., 2000). In compatible (or corresponding) conditions, both routes activate the same (i.e., the correct) response, which is therefore quickly selected and executed. In contrast, in incompatible (or noncorresponding) conditions, the two routes activate different responses, and a response conflict delays selection of the correct response, or sometimes causes an error.

### Compatibility between physical stimulus size and response location

The compatibility effect between physical stimulus size and response location is another compatibility effect that rests on structural overlap between stimuli and responses. It bears some similarity with the SNARC effect, but has received much less attention so far. In a first demonstration, Ren et al. (2011) presented their participants with sequences of two stimuli varying in size, and asked them to indicate whether the second stimulus was smaller or larger than the first by pressing a left or right key. They observed that right-hand responses were faster to large than to small stimuli, while left-hand responses showed a numerical trend in the opposite direction. Wühr and Seegelke (2018) extended this finding in several ways. Firstly, Wühr and Seegelke demonstrated that the effect also occurred in absolute judgments: Pressing a left-hand key to a small stimulus and a right-hand key to a large stimulus (compatible mapping) produced superior performance than the opposite (i.e., incompatible) mapping. Secondly, this compatibility effect was significant for both hands. Thirdly, in a second experiment, Wühr and Seegelke showed that the compatibility effect between physical stimulus size and left/right response location can also occur when stimulus size was task-irrelevant, and participants responded to stimulus color instead (see Richter & Wühr, 2022, for similar results). The latter finding, resembling the Simon effect, suggests that the processing of physical stimulus size, and the activation of associated response codes, occurs in an automatic fashion.

There are two accounts for the structural overlap between physical stimulus size and left/right responses: ATOM (Walsh, 2003, 2015) and polarity correspondence (Proctor & Cho, 2006; Proctor & Xiong, 2015). In *A Theory of Magnitude (ATOM)*, Walsh (2003, 2015) postulated that humans possess a generalized magnitude system that is responsible for the shared representation and processing of space, time, and quantity. Moreover, he assumes that the same metric, a monotonic mapping, is used to mentally represent and process different domains, which is why “bigger, faster, brighter, further in one domain should correlate with bigger, faster, brighter, further in another” (Walsh, 2015, p. 557). Consequently, according to *ATOM*, physical size and space share the same metric. It thus provides a reasonable explanation for the structural overlap between physical size and space, which in turn can account for the automatic associations between stimulus size and response location and thus for the compatibility effect.

The polarity-correspondence principle (Proctor & Cho, 2006; Proctor & Xiong, 2015) holds that, in many binary classification tasks, participants inadvertently assign polarities to alternative stimuli and alternative responses. In particular, when stimuli and responses in a classification task vary on a dimension, and may therefore be conceived as opposite poles on that dimension, participants will assign negative polarity to one stimulus and response, and positive polarity to the other stimulus and responses. This may occur when stimuli and responses vary on different dimensions. During the classification task, the correspondence between stimulus and response polarities may then facilitate performance, whereas their non-correspondence may impair performance, as assumed in dual-route models (e.g., Lakens, 2012; Proctor & Cho, 2006; Wühr & Heuer, 2021). Proctor and Cho (2006) proposed an explanation of the SNARC effect in terms of polarity correspondence (see also Ben Nathan et al., 2009; Shaki et al., 2012; but see Santiago & Lakens, 2015, for conflicting evidence), and a similar explanation could be proposed for the compatibility effect between physical stimulus size and response location. According to this account, participants assign negative polarity to both the small stimulus and the left response, and positive polarity to both the large stimulus and the right response (see Lakens, 2012, and Proctor & Cho, 2006, for evidence supporting these assumptions).

### Absolute and relative stimulus size in visual perception

The main question of our study is whether a correspondence effect between physical object size and response location depends on absolute or relative object size. Absolute size is independent of the size of other objects in a situation (i.e., scene or task), and can be measured in physical units. Relative size, in contrast, describes the size of an object in comparison to other objects in a situation. If, for example, three apples of different size are lying on a table, each apple could be described in terms of absolute or relative size. In everyday situations, our visual field is typically crowded with many objects, rather than containing a single object only. Hence, one might ask whether absolute size does actually play a role for human behavior at all. The answer is positive, however, because absolute object size is an important parameter for controlling our movements (see Jeannerod et al., 1995, for a review). Although research has shown that relative numerical size is more important for the SNARC effect than absolute numerical size, this pattern may not generalize to physical size because even similar correspondence effects may reflect independent adaptations of the human sensorimotor system, and may therefore differ in nature.

According to the influential theory of two visual systems, vision has two quite different tasks, namely “vision for perception” and “vision for action,” and these two tasks are performed by separate visual pathways (Goodale & Milner, 1992; Goodale, 2014; Milner & Goodale, 2006, 2008). “Vision for perception” is performed by a ventral system, leading from primary visual cortex (V1) into temporal cortex. “Vision for action” is performed by a dorsal system, leading from V1 into parietal cortex (cf. Milner & Goodale, 2006; Ungerleider & Mishkin, 1982). The primary task of the ventral system is, according to Milner and Goodale (2006, 2008), the identification of potential target objects for action, and to select an appropriate action plan (vision for perception). In contrast, according to Milner and Goodale (2006, 2008), the primary task of the dorsal system is the implementation of selected action plans by means of visuo-motor control processes (vision for action). A wealth of neurophysiological, neuropsychological, and behavioral data supports the anatomical and functional distinction between the two visual systems (for reviews, see Goodale, 2014; Milner & Goodale, 2006).

A central tenet of the two-visual-systems theory is that the two systems use different spatial reference systems and different metrics. In particular, the ventral system is assumed to process visual objects by relative metrics and in allocentric spatial coordinates, whereas the dorsal system is assumed to process visual objects by absolute metrics and in egocentric (i.e., effector-related) spatial coordinates (e.g., Foley et al., 2015; Goodale, 2014; Milner & Goodale, 2008). That is, the ventral system mainly represents the relative size of an apple, and uses this information for selecting a goal of action, whereas the dorsal system then uses the absolute size of the apple for controlling a grasping movement towards the apple.

Studies investigating the impact of visuo-spatial illusions on perceptual judgments and grasping movements have provided evidence for two visual systems’ use of different metrics. Several studies have demonstrated that irrelevant contextual stimuli can affect perceptual judgments of the size of target objects, whereas the same contextual stimuli do not affect the adaptation of grasping movements to target size (e.g., Aglioti et al., 1995; Cesanek et al., 2018; Ganel et al., 2008; Stöttinger et al., 2010, 2012; Stöttinger & Perner, 2006). For example, Ganel et al. (2008) compared the impact of the Ponzo illusion on perceptual judgments with the impact of this illusion on grasping movements. In the perceptual task, participants reported the perceived length of a line by matching the distance between the thumb and the index finger of their right hand. These judgments were affected by contextual stimuli (reflecting the Ponzo illusion) in that an actually shorter line was judged longer than an actually longer line. In the grasping task, participants “grasped” the line and authors measured the maximal grip aperture (MGA) during the grasping movement, which linearly covaries with object size (Jeannerod, 1986; Marteniuk et al., 1990). Interestingly, the MGA was not affected by the Ponzo illusion, but covaried with actual line length (see also Cesanek et al., 2018). Similar dissociations in the impact of perceptual illusions on perceptual judgments of object size and grasping movements have been reported for other visuo-spatial illusions (e.g., Aglioti et al., 1995; Stöttinger et al., 2010, 2012). These results support the assumption that “vision for perception” processes objects in relative metrics, whereas “vision for action” processes objects in absolute metrics (e.g., Foley et al., 2015; Goodale, 2014; Milner & Goodale, 2008).

If the visual system processes both the relative size and the absolute size of visual objects, then the question arises whether relative or absolute size dominates the correspondence effect between physical object size and response location. Importantly, the characteristics and demands of the participants’ task are a major determinant of whether object size is primarily processed by the ventral or the dorsal system (e.g., Foley et al., 2015; Goodale, 2014). In the present task, stimulus size is completely irrelevant when participants have to report the color of the stimulus object by pressing an appropriate key. Thus, in the present task, stimulus size might be processed along with stimulus color during ventrally dominated stimulus identification, whereas stimulus size does not play a role in (the dorsally mediated) control of the keypress movement. Hence, one might expect that the ventral processing of stimulus size is dominating this task, which occurs in a relative metric according to the two-visual-systems theory (Goodale, 2014).

### The present study

For investigating whether absolute or relative size dominates the correspondence effect between physical stimulus size and left/right responses, we adapted the design used by Dehaene et al. (1993, Experiment 3). In our experiments, participants responded to the color (Experiment 1) or shape (Experiment 2) of a stimulus by pressing a left or right key. In each experimental block, the physical size of the stimuli varied on three levels. Moreover, there were two sets of stimulus sizes, a set of smaller sizes (e.g., 5, 15, 25 mm), and a set of larger sizes (e.g., 25, 35, and 45 mm), which shared the intermediate size (i.e., 25 mm). If absolute size is responsible for the correspondence effect, we should observe different patterns of correspondence effects in the two stimulus-size sets. For example, the left response should be superior within the set of smaller sizes as compared to the set of larger sizes, whereas the right response should be superior within the set of larger sizes as compared to the set of smaller sizes. Moreover, both sets should reveal similar correspondence effects for the shared (i.e., intermediate) size level. In contrast, if relative size is responsible for the correspondence effect, we should observe similar patterns of correspondence effects in the two stimulus-size sets. In particular, within both stimulus sets, the left response should be superior with the small stimulus, both responses should show similar performance for the intermediate size, and the right response should be superior with the large stimulus. Hence, the shared, intermediate stimulus size should show opposite correspondence effects in the two sets.

Addressing the question of whether absolute or relative size dominates the correspondence effect is interesting for several reasons. Firstly, in terms of the two-visual-systems theory (Goodale & Milner, 1992; Goodale, 2014), we are addressing the question whether ventral or dorsal processing of object size is dominating the correspondence effect. Secondly, the results allow for comparisons between different sorts of compatibility effects, and for discovering similarities or differences between effects. As mentioned above, ATOM claims the existence of a generalized magnitude system that uses a common metric for the processing of space, time, quantity, and all sorts of magnitude (e.g., Walsh, 2003, 2015). The assumptions of a shared representation and a common metric seem to suggest similarities between those compatibility (or correspondence) effects that are predicted by ATOM. In other words, shared representations and a common metric seem consistent with the hypothesis that all possible compatibility effects between space, time and different magnitudes rely either on absolute or on relative values.

## Experiment 1

The purpose of Experiment 1 was to investigate whether the correspondence effect between physical stimulus size and left/right responses depends on the stimulus’ absolute or relative size. Therefore, participants performed in a color-discrimination task that required them to press a key to the color (red/green) of a filled square with the left or right hand. Stimulus size varied as an irrelevant stimulus feature, thus leading to corresponding (small-left; large-right) and non-corresponding (small-right; large-left) conditions. Moreover, two stimulus sets were used in different parts of the experiment: A set of smaller stimuli with sizes of 5, 15, and 25 mm, and a set of larger stimuli with sizes of 25, 35, and 45 mm. The intermediate stimulus size of 25 mm was shared by both sets.

We expected to observe a correspondence effect between stimulus size and response location: The left response should be faster and more accurate to smaller stimuli than to larger stimuli, whereas the right response should be faster and more accurate to larger stimuli than to smaller stimuli (e.g., Richter & Wühr, 2022; Wühr & Seegelke, 2018). Moreover, we were interested in whether the pattern of correspondence effects was similar or different with the two stimulus sets. If absolute stimulus size was responsible for the correspondence effect, we should observe different patterns of correspondence effects in the two sets, but similar correspondence effects for the intermediate stimulus size shared by both sets. This pattern should produce a significant three-way interaction of stimulus size, response location, and stimulus set. In contrast, if relative stimulus size was responsible for the correspondence effect, we should observe similar patterns of correspondence effects in the two sets, but different correspondence effects for the shared intermediate size. A prototypical finding would be X-shaped correspondence effects with both sets, and the three-way interaction should not be significant.

Participants also performed in a size-discrimination task before the color-discrimination task. In this task, participants were presented with pairs of stimuli of adjacent size, and responded with the key on the side of the larger stimulus. The size-discrimination task served two purposes. Firstly, we wanted to check that participants were able to discriminate between adjacent sizes, and to compare size-discrimination performance for the two stimulus sets. Secondly, the size-discrimination task also served to remove an ambiguity in the perception of our stimuli. In particular, in our two-dimensional displays, size differences between stimuli could either be perceived as a difference in size or as a difference in distance. A size-discrimination task at the beginning of the experiment should remove this ambiguity.

### Methods

#### Participants

In a previous study, we observed a very strong correspondence effect between irrelevant stimulus size and response location with ${\eta}_p^2$ = .36 (Wühr & Seegelke, 2018, Experiment 2). Since we introduced a new variable (i.e., stimulus set) to the design, we decided to use a smaller effect size of .18 for a power analysis. The program MorePower (Campbell & Thompson, 2012) revealed that a sample size of 50 would suffice to detect an effect of ${\eta}_p^2$ = .18 with high power (1-*β* = .90) in a three-way interaction, with α = .05.

Forty-eight volunteer students (41 female, seven male) with a mean age of 21.35 (*SD* = 2.61) years participated in our experiment and received either course credit or a payment of 5 Euro in exchange. According to self-report, all participants were right-handed, had normal or corrected-to-normal vision, and normal color vision. Prior to participation, all volunteers gave their informed consent. The local Ethics Committee at TU Dortmund University had approved the experimental protocol for our study (2018-09).

#### Apparatus and stimuli

Participants sat in front of a 17-in. color monitor of a customary computer, with a viewing distance of approximately 50 cm. A computer program, written with E-Prime 2.0 (Psychology Software Tools; Sharpsburg, PA, USA) controlled the presentation of stimuli and registered responses (i.e., key pressed, reaction time (RT)). A small plus sign (Courier font, size 18 pt), which was presented at the screen center at the beginning of each trial, served as a fixation point. As imperative stimuli, five red and green squares of different sizes varying between 5 mm and 45 mm in increments of 10 mm were presented on a gray background (E-Prime color “silver”) at the center of the screen.^2^ From a distance of 50 cm, the five stimuli subtended visual angles of approximately 0.573°, 1.719°, 2.864°, 4.009°, and 5.153°. Ten distinct imperative stimuli were employed, resulting from the orthogonal combination of two colors (red/green) and five sizes. Participants responded to the stimuli by pressing the left Control key or the right Enter key of a standard keyboard with the index fingers of their left and right hand, respectively. The keyboard was centrally aligned to the participants’ midline. To avoid ambiguity, both relevant keys were additionally marked with black tape.

#### Procedure

In this experiment, participants completed a size-discrimination task with two stimulus sets, and a color-discrimination task with two stimulus sets. The set of smaller stimuli contained three squares of 5 mm, 15 mm and 25 mm. The set of larger stimuli contained three squares of 25 mm, 35 mm and 45 mm. The size-discrimination task with a particular stimulus set was always administered before the color-discrimination task with the same set. The order of stimulus sets, however, was counterbalanced across participants, resulting in 24 participants with the small-large order, and 24 participants with the large-small order.

**Fig. 1:**
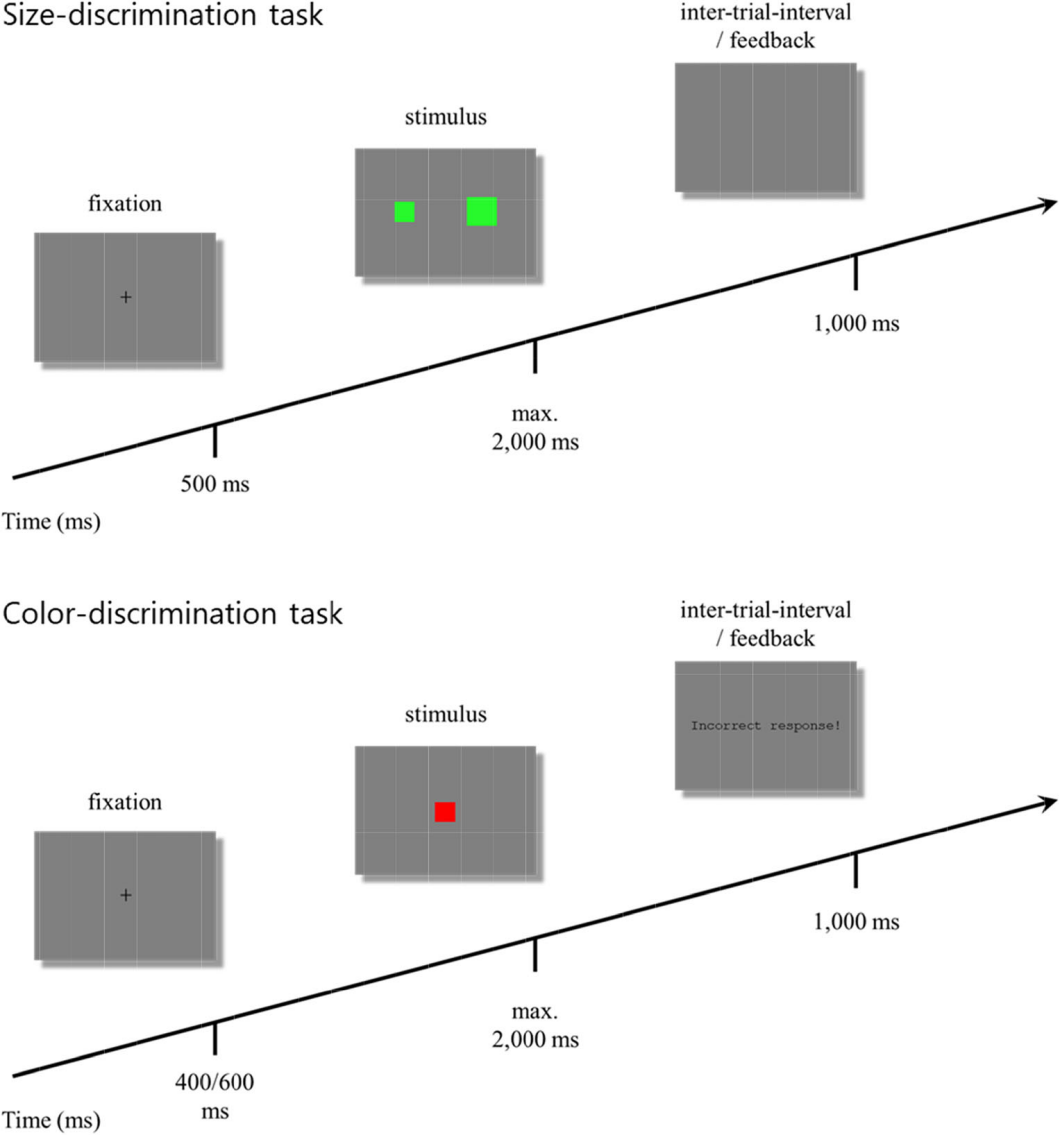
Time-course of events in typical trials of the size-discrimination task (upper panel), and the color-discrimination task (lower panel)

In the size-discrimination task, two stimuli of adjacent size were shown on the screen, one to the left and the other to the right of fixation. Participants were to press the key on the side of the larger stimulus of each pair. The size-discrimination task consisted of only one block with 40 trials. The number of trials resulted from orthogonally combining two pairs of sizes (e.g., 1-2, 2-3), two spatial configurations (small-large, large-small), two colors (green, red), and five repetitions. A trial started with a fixation point for 500 ms, followed by the presentation of a stimulus pair until a response was made, or until a maximal presentation time of 2,000 ms had elapsed. An inter-trial interval of 1,000 ms with an empty screen followed each correct response. In contrast, after an error or if no response had been made, a corresponding error message was presented during the inter-trial interval. The time course and sample stimuli of the size-discrimination task are depicted in Fig. 1.

In the color-discrimination task, participants were to respond to the red/green color of a square by pressing the left/right key with their left/right index finger, respectively. The mapping between color as relevant stimulus feature and response location was counterbalanced across participants. For each of the two stimulus sets, the color-discrimination task included one training block and five experimental blocks. The training blocks consisted of 12 trials, and the experimental blocks consisted of 48 trials, respectively. Within each block, trials occurred in random order. At the beginning of each task, instructions presented at the screen informed participants about the content and the procedure of the following task. At the beginning of each trial, a fixation point was presented for 400 or 600 ms, with both durations occurring equally often within each block. The imperative stimulus was then presented until a response was made or for a maximum of 2,000 ms. As for the size-discrimination task, an inter-trial interval of 1,000 ms with an empty screen followed each correct response. In contrast, after an error or if no response had been made, a corresponding error message was presented during the inter-trial interval. The time course and sample stimuli of the color-discrimination task are depicted in Fig. 1. Participants were free to take a break between blocks or to continue with the subsequent one. The experiment took about 30 min. The experimenter stayed in the laboratory for the practice blocks and left the room before participants started the first experimental block.

#### Design

We planned to separately analyze the results from the two tasks. Concerning the color-discrimination task, we planned to analyze the effects of three independent variables on RTs and error percentages in two 3 (Stimulus Size) × 2 (Response Location) × 2 (Stimulus Set) analyses of variance (ANOVAs). We manipulated all three independent variables within participants. The factor stimulus set contained two levels: a set of smaller stimuli and a set of larger stimuli. Within each set, stimulus size was varied on three levels. Using three stimulus values in each set gives us the opportunity for testing whether the correspondence effect is actually absent for the intermediate stimulus size within each set, which could be expected if relative size was important. The factor response location had two levels, specifically left versus right response location. For all ANOVAs involving the three-level factor Stimulus Size, we performed a Greenhouse-Geisser correction of the degrees of freedom, if Mauchly’s test of sphericity was significant.

Concerning the size-discrimination task, we planned to analyze the effect of Stimulus Set on RTs and error percentages in this task. Note that the main purpose of this task was to check if participants were able to discriminate safely between adjacent levels of stimulus size, and whether this ability would differ between stimulus sets.

### Results

#### RTs in color-discrimination task

A three-factorial repeated-measures ANOVA was conducted with *Stimulus Size*, *Response Location*, and *Stimulus Set* as independent within-subjects variables, and RT as dependent variable. The corresponding cell means are depicted in Table 1. A significant main effect of *Stimulus Size*, *F*(1.667, 78.344) = 22.244, *MSE* = 198.986, *p* < .001, ${\eta}_p^2$ = .321, revealed shorter RTs for larger stimuli (level 3: *M* = 372 ms, *SD* = 46) than for smaller stimuli (level 1: *M* = 379 ms, *SD* = 44). The main effects of *Response Location*, *F*(1, 47) = 1.689, *MSE* = 1078.074, *p* = .20, ${\eta}_p^2$ = .035, and *Stimulus Set*, *F*(1, 47) = 0.038, *MSE* = 1555.237, *p* = .847, ${\eta}_p^2$ = .001, were not significant. However, a significant two-way interaction between *Stimulus Size* and *Response Location*, *F*(2, 94) = 4.876, *MSE* = 206.272, *p* = .01, ${\eta}_p^2$ = .094, signaled the presence of a correspondence effect (see below). In addition, the correspondence effect was not affected by *Stimulus Set*, because the three-way interaction between *Stimulus Size*, *Response Location* and *Stimulus Set* was far from significance, *F*(2, 94) = 1.171, *MSE* = 271.038, *p* = .315, ${\eta}_p^2$ = .024. Further results included a significant two-way interaction between *Stimulus Size* and *Stimulus Set*, *F*(2, 94) = 8.080, *MSE* = 254.307, *p* < .001, ${\eta}_p^2$ = .147, with faster responses to the large stimulus size in the small stimulus set and to the small stimulus size in the large stimulus set, as well as a non-significant interaction between *Response Location* and *Stimulus Set*, *F*(1, 47) = 0.402, *MSE* = 282.923, *p* = .529, ${\eta}_p^2$ = .008.

**Table 1:** Mean corrected reaction times (RTs, ms) observed in Experiment 1 as a function of Stimulus Set, Response Location, and Stimulus Size (1, 2, 3)

	Set of Smaller Stimuli			Set of Larger Stimuli		
	1	2	3	3	4	5
Left Response	379 (37)	370 (40)	373 (48)	374 (44)	373 (43)	376 (40)
Right Response	388 (51)	368 (45)	369 (44)	376 (43)	373 (45)	372 (50)

Standard errors are given in parentheses

If we consider only those stimulus values that were shared by the two stimulus sets, the numerical pattern fitted the predictions of the relative-size-counts hypothesis. The shared stimulus was the largest one in the set of smaller sizes, and therefore right responses were numerically faster than left responses, whereas the opposite was observed for the set of larger sizes (cf. Table 1). However, the corresponding *Response Location* × *Stimulus Set* interaction was not significant, *F*(1, 47) = 1.446, *MSE* = 206.831, *p* = .235, ${\eta}_p^2$ = .030.

**Fig. 2:**
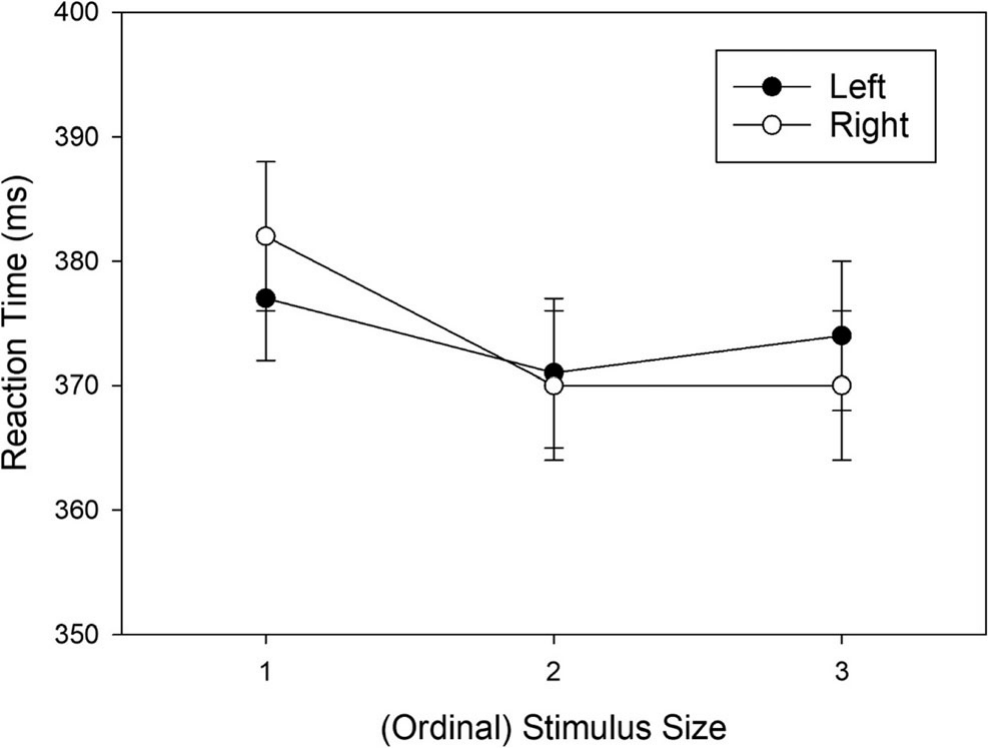
Mean reaction times (RTs, ms) observed in Experiment 1 as a function of ordinal Stimulus Size (collapsed across stimulus sets) and Response Location (main effect of response removed from data). Error bars represent standard errors of the mean (between participants)

Because the three-way interaction was not significant in the omnibus analysis, we collapsed the data across stimulus sets, and further explored the source of the Stimulus Size × Response Location interaction (i.e., the correspondence effect) by comparing RTs of left responses to RTs of right responses for each level of stimulus size. Even though the main effect of response location was non-significant, we removed the numerical effect from the data (Harwell, 1998; Rosnow & Rosenthal, 1989). The adjusted means of the resulting conditions are shown in Fig. 2. Shapiro-Wilk tests revealed that the distribution of the pair-wise differences deviated from normal for two out of three cases, and therefore we used Wilcoxon’s signed-rank test for pair-wise comparisons. For the small stimulus size 1, the numerical advantage for left over right responses was not significant, *W*(47) = 441, *p* = .134, *r*_*rb*_ = -.250. A numerical advantage for right over left responses was neither significant for the intermediate stimulus size 2, *W*(47) = 610, *p* = .827, *r*_*rb*_ = .037, nor for the large stimulus size 3, *W*(47) = 699, *p* = .260, *r*_*rb*_ = .189.

#### Error percentages in color-discrimination task

The error percentages were also subjected to a three-factorial repeated-measures ANOVA with *Stimulus Size*, *Response Location*, and *Stimulus Set* as independent variables. The means are depicted in Table 2. The main effects of *Stimulus Size*, *F*(1.721, 80.886) = 1.627, *MSE* = 8.962, *p* = .206, ${\eta}_p^2$ = .033, and *Stimulus Set*, *F*(1, 47) = 0.806, *MSE* = 5.938, *p* = .374, ${\eta}_p^2$ = .017, were not significant. However, a significant main effect of *Response Location* occurred, *F*(1, 47) = 5.868, *MSE* = 7.812, *p* = .019, ${\eta}_p^2$ = .111, resulting from a smaller error percentage for left-hand (*M* = 2.318, *SD* = 2.695) than right-hand (*M* = 2.882, *SD* = 3.100) responses. A significant two-way interaction of *Stimulus Size* and *Response Location*, *F*(2, 94) = 7.140, *MSE* = 9.562, *p* = .001, ${\eta}_p^2$ = .132, indicated the presence of a correspondence effect (see below). Importantly, and consistent with the RT results, the correspondence effect was independent from *Stimulus Set*, because the three-way interaction was not significant, *F*(1.678, 78.865) = 0.696, *MSE* = 6.768, *p* = .478, ${\eta}_p^2$ = .015. Neither the *Stimulus Size* × *Stimulus Set*, *F*(2, 94) = 1.673, *MSE* = 7.084, *p* = .193, ${\eta}_p^2$ = .034, nor the *Response Location* × *Stimulus Set* interaction, *F*(1, 47) = 0.648, *MSE* = 6.045, *p* = .425, ${\eta}_p^2$ = .014, was significant.

**Table 2:** Mean corrected error percentages (%) observed in Experiment 1 as a function of Stimulus Set, Response Location, and Stimulus Size

	Set of Smaller Stimuli			Set of Larger Stimuli		
	1	2	3	3	4	5
Left Response	2.209 (2.789)	2.470 (2.759)	3.147 (2.824)	1.949 (2.268)	2.522 (2.377)	3.303 (2.961)
Right Response	4.041 (2.996)	2.062 (3.025)	2.218 (3.137)	2.791 (3.469)	2.166 (2.801)	2.322 (2.823)

Standard errors are given in parentheses

If we consider only those stimulus values that are shared by the two stimulus sets, the numerical pattern again fits the predictions of the relative-size-counts hypothesis. The shared stimulus is the largest one in the set of smaller sizes, and therefore right responses are more accurate than left responses, whereas the opposite holds for the set of larger sizes (cf. Table 2). This pattern led to a significant two-way interaction, *F*(1, 47) = 4.839, *MSE* = 7.776, *p* = .033, ${\eta}_p^2$ = .093.

**Fig. 3:**
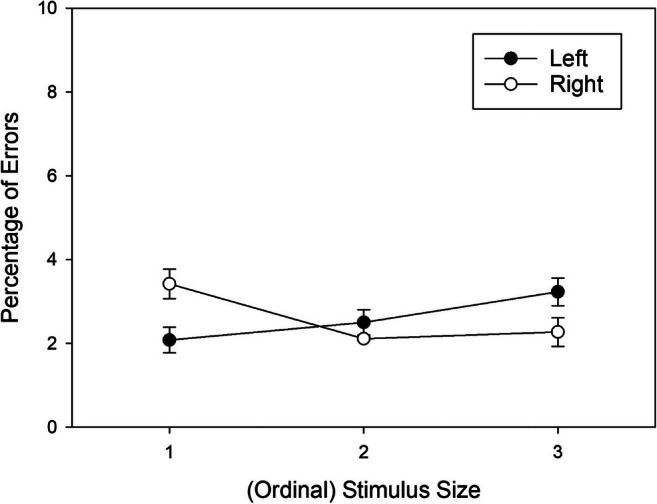
Mean error percentages (%) observed in Experiment 1 as a function of ordinal Stimulus Size (collapsed across stimulus sets) and Response Location (main effect of response removed from data). Error bars are standard errors between participants

Due to the non-significant three-way interaction in the omnibus analysis, we collapsed the data across stimulus sets, and further explored the source of the Stimulus Size × Response Location interaction (i.e., the correspondence effect) by comparing error percentages in left responses to error percentages in right responses for each level of stimulus size. We corrected the data for the significant main effect of response location. The adjusted means of the resulting conditions are shown in Fig. 3. Shapiro-Wilk tests revealed that the distribution of the pair-wise differences deviated from normal for the intermediate stimulus size, and we therefore used Wilcoxon’s signed-rank test for pair-wise comparisons. For the small stimulus size 1, the error percentage was significantly lower for left responses as compared to right responses, *W*(47) = 255, *p* < .001, *r*_*rb*_ = -.566. For the intermediate stimulus size 2, error percentages for left and right responses did not differ, *W*(47) = 621, *p* = .738, *r*_*rb*_ = .056. For the large stimulus size 3, the advantage for right over left responses was marginally significant, *W*(47) = 756, *p* = .085, *r*_*rb*_ = .286.

#### Size-discrimination task

We analyzed performance in the size-discrimination task by comparing RTs and error percentages between the two stimulus sets. Discrimination RTs were significantly shorter with the set of smaller stimuli (*M* = 377, *SD* = 52) than with the set of larger stimuli (*M* = 401, *SD* = 62), *W*(47) = 259, *p* < .001, *r*_rb_ = -.411. The percentage of discrimination errors was numerically smaller with the set of smaller stimuli (*M* = 0.990, *SD* = 1.609) than with the set of larger stimuli (*M* = 1.563, *SD* = 2.342), but the difference was not significant, *W*(47) = 68, *p* = .083, *r*_rb_ = -.411. Better discrimination performance for the small set as compared to the large set is a consequence of the fact that size-discrimination performance improves when the ratio of to-be-compared sizes increases (e.g., Leibovich et al., 2013), as predicted by Weber’s law (cf., Pardo-Vazquez et al., 2019). Due to the constant increase in diameter, the size ratios of to-be-compared stimuli were larger for the set of smaller stimuli than for the set of larger stimuli. Alternatively, the increase of RTs with increasing stimulus size might be related to an inverse relationship between the size of the attentional window and attentional resolution within that window (e.g., Castiello & Umiltà, 1990; Eriksen & St. James, 1986).

### Discussion

The results of Experiment 1 revealed a correspondence effect between physical stimulus size and left/right responses both in RTs and in error percentages. The left response was numerically faster and more accurate to small stimuli than the right response, whereas the right response was numerically faster and more accurate to large stimuli than the left response, replicating previous findings (i.e., Richter & Wühr, 2022; Wühr & Seegelke, 2018). Most importantly, similar patterns of correspondence effects were observed with two different stimulus sets. The similarity of correspondence effects with the two stimulus sets provides first evidence that relative, and not absolute, stimulus size is involved in the correspondence (and compatibility) effect between physical stimulus size and left/right responses.

Two results of Experiment 1 were dissatisfying, however. Firstly, although a significant two-way interaction in RTs signaled the presence of a correspondence effect across stimulus sets, the pair-wise comparisons for individual size levels were not significant. Secondly, we observed an unpredicted main effect of stimulus size on RTs in the color-discrimination task: responses were faster with larger stimuli than with smaller stimuli. We assume this main effect reflects better color discrimination performance with larger stimuli than with smaller stimuli, consistent with previous findings (e.g., Brown, 1952; Nagy, 1994). Previous research has shown that spatial correspondence effects, such as the Simon effect, vary with RT level (e.g., Hommel, 1994; see, Proctor et al., 2011, for a review), and therefore we cannot exclude that the main effect (of stimulus size) on RTs may have also affected the magnitude of the correspondence effect. Therefore, we sought to replicate our main findings while avoiding a main effect of stimulus size.

## Experiment 2

The main purpose of Experiment 2 was to replicate the main findings from Experiment 1, which consisted of similar correspondence effects between physical stimulus size and left/right responses for two sets of different stimulus sizes, while avoiding a main effect of stimulus size in the color-discrimination task. The basic design of Experiment 2 was similar to Experiment 1, but there were three notable differences. Firstly, we changed the main task from a color-discrimination task to a shape-discrimination task. Hence, in Experiment 2, participants had to press one key if the stimulus was a circle and another key if the stimulus was a square. We changed the relevant stimulus feature because a main effect of stimulus size in Experiment 1 suggested that color discrimination became more difficult when size decreased, and we feared that variations in the difficulty of color-discrimination performance might also affect the correspondence effect.^3^ Secondly, because the COVID-19 pandemic prevented testing participants in our laboratory, the data for Experiment 2 were collected at participants’ homes on their private computers (see *Method* section for more details). Thirdly, because pilot testing had revealed that stimuli were slightly downscaled on laptop (not desktop) computers, we decided to increase stimulus sizes while keeping size differences constant. Hence, in Experiment 2, one stimulus set contained stimuli of 20, 30, and 40 mm in size, whereas the other set contained stimuli of 40, 50, and 60 mm in size.

### Methods

#### Participants

We aimed for the same sample size as in Experiment 1 (i.e., 50). Because the Coronavirus pandemic prevented data collection in our laboratory, we decided to conduct this study as a home experiment. We advertised our experiment on an online platform for students of TU Dortmund University, and 70 students (54 females, 16 males; average age = 23.15 years) replied to our advertisement. Participation in this experiment required that participants had an IBM-compatible PC (or laptop computer) with Windows 10 as the operating system. We sent the computer programs for conducting the experiment, and a text file with instructions, to the students who had replied to our call for participation.

From the 70 students who had originally consented to participate, 12 did not send back any data files. From the remaining 58 participants, we excluded seven participants because they were left-handers. Hence, the final sample of participants included 51 participants (45 female, six male) with a mean age of 23.16 (*SD* = 4.51) years. They received course credit in exchange. All participants in the final sample gave informed consent, reported being right-handed, and having normal or corrected-to-normal vision.

#### Apparatus and stimuli

Experiment 2 was conducted as a home experiment using the software E-Prime Go in combination with the software E-Prime 3.0 (Psychology Software Tools; Sharpsburg, PA, USA). E-Prime Go converts programs written with E-prime 3.0 into programs that are executable on any IBM-compatible computer, which runs under the operating system Windows 10 (Microsoft Corporation; Redmond, WA, USA). Participants could use the program “Pc Qualify” to check if their computer fulfilled the technical requirements for our experiment. To establish similar viewing conditions, participants were instructed to set their screen resolution to 1,920 × 1,080 and the scaling to 100%. E-Prime Go controlled the timing of events, the presentation of stimuli, collected technical variables (e.g., screen refresh rate, screen resolution), and measured response parameters (e.g., pressed keys, RTs of keypress responses). These measurements and parameters were written into data files, which participants sent back to us after having finished the experiment.

As in Experiment 1, a fixation point (plus sign in Courier font, size 18 pt) was presented at the screen center at the beginning of each trial. The imperative stimuli used in Experiment 2 were similar to the ones in Experiment 1 with the exception that instead of stimulus color (red/green), the outline shape of the stimulus, which was either a square or a circle, served as the relevant stimulus feature. The size of the stimuli, i.e., the side length of a square, or the diameter of a circle, varied as an irrelevant feature between 20 mm and 60 mm in increments of 10 mm. Since six different sizes were orthogonally combined with two shapes, a total of 12 distinct imperative stimuli were used and presented on a gray background (E-Prime color “silver”) at the center of the screen. Participants had to respond by pressing the left Control key or the right Enter key of a standard keyboard with the index fingers of their left and right hand.

#### Procedure

Participants were provided with the necessary E-Prime Go files via an online course platform. They were asked to execute the program in a quiet environment whenever they had enough time. The procedure of Experiment 2 was similar to that of Experiment 1 with the following exceptions. Firstly, we dropped the size-discrimination task from the experiment. Instead, we inserted a short block during the instructions, in which participants were shown each stimulus and were asked to measure the size of the stimulus. Participants had to measure the side length of a square, or the diameter of a circle, with a ruler, and type the result into the computer. Secondly, instead of stimulus color, stimulus shape (square/circle) served as the relevant stimulus feature in Experiment 2. In particular, the experiment consisted of two parts (each implemented by a separate computer program). In one part, participants performed the shape-discrimination task with a set of small stimuli (20, 30, 40 mm); in the other part, participants performed the shape-discrimination task with a set of large stimuli (40, 50, 60 mm). The order of stimulus sets as well as the mapping between shape as relevant stimulus feature and response location were counterbalanced across participants. Each part included a practice block of 24 trials, and four experimental blocks of 48 trials. After finishing the experiment, participants sent the two output-files created by E-Prime Go to the experimenter.

#### Design

Similar to Experiment 1, Experiment 2 had a 3 (Stimulus Size) × 2 (Response Location) × 2 (Stimulus Set) within-subjects design. The factor stimulus set comprised a set of smaller stimuli with sizes between 20 and 40 mm, and a set of larger stimuli with sizes between 40 and 60 mm. In turn, within each stimulus set, three sizes of stimuli were employed: a small, intermediate and large stimulus. Response location included left- versus right-hand responses. Reaction times of correct responses (RT) and error percentages (EP) were measured as dependent variables.

### Results

#### Size-measurements

Each participant provided 12 measurements of stimulus size (6 sizes × 2 shapes). It turned out that the actual sizes of the stimuli on their computer screens varied despite the fact that all used the same pictures, the same programs, the same operating system, and the same monitor settings (i.e., resolution, and scaling). Hence, we decided to include the data from all participants whose size measurements increased as a strictly monotonous function of actual size, and when the sizes for corresponding stimuli of different shapes were similar (i.e., did not differ by more than 5 mm). The results of these measurements are provided in Table 3. These results demonstrate that actual stimulus size was, on average, by 25% smaller than the desired stimulus size. As a result, the actual increment was, on average, 7.5 mm instead of the desired value of 10 mm.

**Table 3:** Results of participants’ measurements of stimulus size (in mm) on their computers. Standard deviations are given in parentheses^a^

	Original Stimulus Size (in mm)				
	20	30	40	50	60
Circle	15.10 (4.69)	22.57 (6.57)	29.98 (9.13)	37.43 (11.66)	44.51 (13.57)
Square	15.51 (4.71)	23.00 (6.53)	30.39 (8.82)	37.89 (11.29)	45.14 (13.09)

^a^We refrain from reporting visual angles for the stimuli used in Experiment 2 because we do not know the viewing distances of our participants, and absolute stimulus sizes also varied between participants

#### Reaction times

RTs were subjected to a three-factorial repeated-measures ANOVA with *Stimulus Size*, *Response Location*, and *Stimulus Set* as within-subjects variables. The corrected means are presented in Table 4. None of the main effects was significant, *Stimulus Size*: *F*(2, 100) = 0.659, *MSE* = 241.286, *p* = .520, ${\eta}_p^2$ = .013, *Response Location*: *F*(1, 50) = 1.492, *MSE* = 1671.519, *p* = .228, ${\eta}_p^2$ = .029, and *Stimulus Set*: *F*(1, 50) = 2.321, *MSE* = 1141.800, *p* = .134, ${\eta}_p^2$ = .044. However, a significant two-way interaction between *Stimulus Size* and *Response Location*, *F*(2, 100) = 10.613, *MSE* = 393.343, *p* < .001, ${\eta}_p^2$ = .175, indicated the presence of the correspondence effect (see below). Most importantly, the three-way interaction between *Stimulus Size*, *Response Location*, and *Stimulus Set* was again not significant, *F*(2, 100) = 1.428, *MSE* = 413.997, *p* = .245, ${\eta}_p^2$ = .028. The two-way interactions *Stimulus Size* × *Stimulus Set*, *F*(2, 100) = 0.795, *MSE* = 335.157, *p* = .454, ${\eta}_p^2$ = .016, and *Response Location* × *Stimulus Set*, *F*(1, 50) = 0.218, *MSE* = 658.499, *p* = .643, ${\eta}_p^2$ = .004, were not significant.

**Table 4:** Mean corrected reaction times (RTs, ms) observed in Experiment 2 as a function of Stimulus Set, Response Location, and Stimulus Size (1, 2, 3)

	Set of Smaller Stimuli			Set of Larger Stimuli		
	1	2	3	3	4	5
Left Response	389 (44)	393 (46)	401 (48)	383 (51)	392 (55)	393 (60)
Right Response	399 (47)	393 (55)	388 (48)	392 (54)	389 (52)	389 (49)

Standard errors are given in parentheses

If we consider only those stimulus values that are shared by the two stimulus sets, the numerical pattern again confirmed the predictions of the relative-size-counts hypothesis. The shared stimulus was the largest one in the set of smaller sizes, and therefore right responses were faster than left responses, whereas the opposite was true for the set of larger sizes (cf. Table 4). This pattern led to a significant *Response Location* × *Stimulus Set* interaction, *F*(1, 50) = 17.546, *MSE* = 359.051, *p* < .001, ${\eta}_p^2$ = .260.

**Fig. 4:**
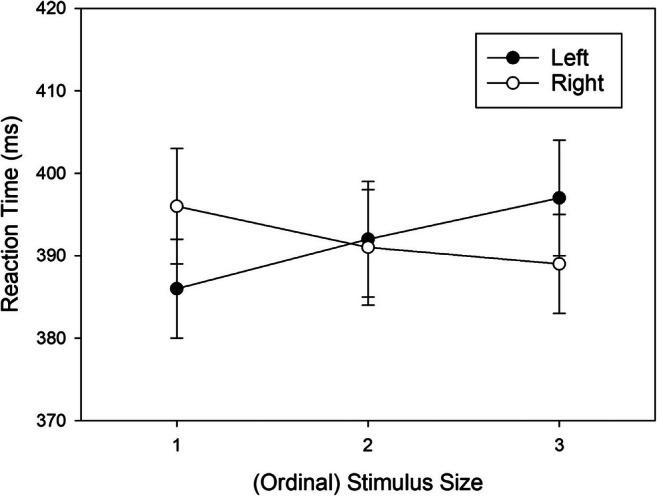
Mean reaction times (RTs, ms) observed in Experiment 2 as a function of ordinal Stimulus Size (collapsed across stimulus sets) and Response Location (main effect of response removed from data). Error bars represent standard errors (between participants)

Due to the non-significant three-way interaction, we collapsed the data across stimulus sets, and further explored the source of the Stimulus Size × Response Location interaction (i.e., the correspondence effect) by comparing RTs of left responses to RTs of right responses for each level of stimulus size. We subtracted the numerical main effect of response location from the data (Harwell, 1998; Rosnow & Rosenthal, 1989). The adjusted means of the resulting conditions are shown in Fig. 4. Shapiro-Wilk tests revealed that the distribution of the pair-wise differences did not significantly deviate from normal, and we therefore used *t* tests for pair-wise comparisons. For the small stimulus size 1, RTs were significantly shorter for left compared to right responses, *t*(50) = -2.622, *p* = .012, *d*_*z*_ = -0.367. For the intermediate stimulus size 2, there was no significant difference between right- and left-hand responses, *t*(50) = 0.420, *p* = .676, *d*_*z*_ = 0.059. Finally, for the large stimulus size 3, RTs were marginally shorter for right as compared to left responses, *t*(50) = 1.930, *p* = .059, *d*_*z*_ = 0.270.

#### Error percentages

A three-factorial ANOVA was conducted with *Stimulus Size*, *Response Location*, and *Stimulus Set* as independent variables, and error percentage as dependent variable. The corresponding means are shown in Table 5. A significant main effect of *Response Location*, *F*(1, 50) = 11.445, *MSE* = 8.702, *p* = .001, ${\eta}_p^2$ = .186, revealed more accurate left-hand responses (*M* = 2.614, *SD* = 3.260) than right-hand responses (*M* = 3.421, *SD* = 3.686). The main effects of *Stimulus Size*, *F*(1.791, 89.573) = 0.765, *MSE* = 12.009, *p* = .455, ${\eta}_p^2$ = .015, and *Stimulus Set*, *F*(1, 50) = 0.101, *MSE* = 7.748, *p* = .752, ${\eta}_p^2$ = .002, were not significant. Crucially, the interaction between *Stimulus Size* and *Response Location* was again significant, *F*(2, 100) = 17.041, *MSE* = 11.690, *p* < .001, ${\eta}_p^2$ = .254, whereas the three-way interaction between *Stimulus Size*, *Response Location*, and *Stimulus Set* was not, *F*(2, 100) = 1.969, *MSE* = 8.847, *p* = .145, ${\eta}_p^2$ = .038. Hence, the results signaled the presence of a correspondence effect that was independent of *Stimulus Set*. Neither the *Stimulus Size* × *Stimulus Set*, *F*(2, 100) = 0.809, *MSE* = 11.676, *p* = .448, ${\eta}_p^2$ = .016, nor the *Response Location* × *Stimulus Set* interaction, *F*(1, 50) = 0.530, *MSE* = 6.780, *p* = .470, ${\eta}_p^2$ = .010, was significant.

**Table 5:** Mean corrected error percentages (%) observed in Experiment 2 as a function of Stimulus Set, Response Location, and Stimulus Size

	Set of Smaller Stimuli			Set of Larger Stimuli		
	1	2	3	3	4	5
Left Response	1.751 (2.188)	3.099 (2.933)	4.080 (4.537)	2.548 (2.688)	3.283 (3.115)	3.345 (3.272)
Right Response	4.682 (4.633)	2.660 (2.829)	2.048 (2.747)	3.825 (4.322)	3.151 (3.803)	1.741 (2.538)

Standard errors are shown in parentheses

If we consider only those stimulus values that are shared by the two stimulus sets, the numerical pattern again confirmed the predictions of the relative-size-counts hypothesis. The shared stimulus was the largest one in the set of smaller sizes, and therefore right responses were more accurate than left responses, whereas the opposite was true for the set of larger sizes (cf. Table 5). This pattern also produced a significant *Response Location* × *Stimulus Set* interaction, *F*(1, 50) = 11.131, *MSE* = 12.540, *p* = .002, ${\eta}_p^2$ = .182.

**Fig. 5:**
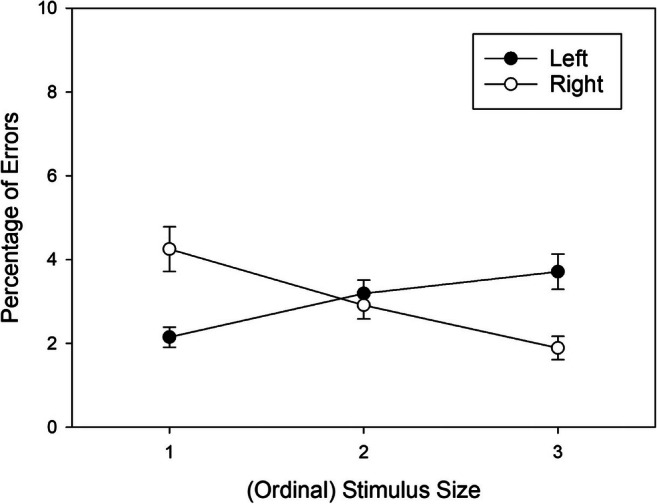
Mean error percentages (%) observed in Experiment 2 as a function of ordinal Stimulus Size (collapsed across stimulus sets) and Response Location (main effect of response removed from data). Error bars are standard errors between participants

Due to the non-significant three-way interaction, we collapsed data across stimulus sets, and further explored the source of the *Stimulus Size* × *Response Location* interaction (i.e., the correspondence effect) by comparing error percentages of left responses to those of right responses for each level of stimulus size. We corrected the data for the significant main effect of response location. The adjusted means of the resulting conditions are shown in Fig. 5. Shapiro-Wilk tests revealed that the distribution of the pair-wise differences deviated from normal for all three size conditions, and we therefore used Wilcoxon’s signed-rank test for pair-wise comparisons. For the small stimulus size 1, the error percentage was significantly lower for left as compared to right responses, *W*(50) = 350, *p* = .003, *r*_*rb*_ = -.472. For the intermediate stimulus size 2, error percentages for left and right responses did not differ, *W*(50) = 844, *p* = .090, *r*_*rb*_ = .273. Finally, for the large stimulus size 3, the error percentage was significantly lower for right as compared to left responses, *W*(50) = 1147, *p* < .001, *r*_*rb*_ = .730.

### Discussion

Experiment 2 successfully replicated the major results of Experiment 1, despite several methodological differences. For two stimulus sets, each containing three stimuli of increasing size, the left response was faster and more accurate than the right response for the small stimulus within each set, whereas the right response was faster and more accurate than the left response for the large stimulus within each set. In both sets, the size-RT functions of the two responses crossed at the intermediate stimulus size. Moreover, the correspondence effects were highly similar with both stimulus sets, as indicated by the absence of a significant three-way interaction, providing corroborating evidence that relative, and not absolute, stimulus size is critical for the correspondence effect between physical stimulus size and left/right responses. The most striking support for this conclusion comes from the fact that opposite correspondence effects were observed for the same stimulus size of 40 mm, which was the largest stimulus in one set but the smallest stimulus in the other set.

The methodological changes between the two experiments worked out as expected. In particular, using shape instead of color as the relevant stimulus feature eliminated main effects of stimulus size in Experiment 2. Hence, shape-discrimination performance was independent of stimulus size, and did not deteriorate when size decreased, as did color-discrimination performance in Experiment 1. This may have also eliminated possible variations in the size of the correspondence effect with RT level, as observed for spatial correspondence effects (e.g., De Jong et al., 1994; Hommel, 1994). Moreover, the fact that changing the relevant discrimination task eliminated the main effect of stimulus size on RTs also supports our interpretation of this main effect as an effect of stimulus size on (the difficulty of) color discrimination (e.g., Brown, 1952; Nagy, 1994).

## General discussion

In two experiments, we investigated whether absolute or relative physical stimulus size is more important for a correspondence effect between physical stimulus size and left/right responses. Therefore, we compared the correspondence effect for two overlapping sets of stimulus sizes, a set of three smaller sizes and a set of three larger sizes. If absolute stimulus size was critical, we should find different patterns of correspondence effects in the two sets, but similar correspondence effects for an intermediate stimulus value shared by the sets. In Experiment 1, participants responded to stimulus color (red or green), and stimulus size varied in steps of 10 mm. In Experiment 2, participants responded to stimulus shape (circle or square), and stimulus size varied in steps of 7 mm. The experiments produced a consistent pattern of results. Significant two-way interactions of stimulus size and response location indicated the presence of the correspondence effect both in RTs and in error percentages. Most importantly, the three-way interaction of stimulus size, response location, and stimulus set was never significant, indicating similar patterns of correspondence effects with different stimulus sets. In fact, X-shaped patterns of correspondence effects occurred in both sets of stimulus sizes. Moreover, the shared, intermediate stimulus size showed an advantage for the right response in the set of smaller stimuli, and an advantage for the left response in the set of larger stimuli. Hence the pattern of results of our experiments strongly supports the hypothesis that relative stimulus size is more important than absolute size for the correspondence effect between physical stimulus size and left/right responses.

### Absolute versus relative size

Our study addressed the question of whether vision for perception (the ventral system) or vision for action (the dorsal system) dominates the processing of object size in our participants’ task, which reveals a correspondence effect between physical stimulus size and left/right responses. According to the influential theory of two visual systems, the ventral system processes visual information for classifying and identifying objects (vision for perception), which represent potential action goals, whereas the dorsal system processes visual information for controlling movements to achieve the selected goals (vision for action; Goodale, 2014; Milner & Goodale, 2006, 2008). A central claim of the theory holds that the ventral system uses a relative metric, whereas the dorsal system uses an absolute metric for processing visual stimuli (cf. Foley et al., 2015; Goodale, 2014; Milner & Goodale, 2008). From the perspective of the two-visual-systems theory our results can thus be interpreted as showing that the ventral system, which serves the classification and identification of visual objects, dominates the processing of irrelevant stimulus size in our experiments.

The interpretation of our results from the viewpoint of the two-visual-systems theory makes sense, if one takes a closer look on the participants’ task used in our experiments. Our task requires a binary classification of stimuli according to color or shape by making an arbitrary keypress response. Hence, stimulus size is an irrelevant stimulus feature that is neither required for stimulus classification nor for the selection or execution of the response. Thus, our task most presumably requires mainly a perceptual classification judgment by the ventral system, and no processing of stimulus size by the dorsal system.

In this context, one may ask why stimulus size is processed at all in our task, although this feature is completely irrelevant for the participants’ task. An answer to this question can be found in research on object-based visual attention, which has shown that attentional selection of a visual object not only amplifies the processing of relevant stimulus features, but also triggers the processing of irrelevant stimulus features of the attended object (e.g., Behrmann et al., 1998; Duncan, 1984; Kahneman & Henik, 1981; Wühr & Waszak, 2003). With regard to the Simon effect, an instance of spatial S-R correspondence, Wühr et al. (2008) showed that the irrelevant location of an attended object produces much stronger spatial correspondence effects than the, equally salient, irrelevant location of an unattended visual object. Hence, we assume that stimulus size is processed in our task because it is a feature of the attended visual object, and size is processed in a relative metric because the ventral system (i.e., vision for perception) dominates stimulus classification in our task.

Although the results of our experiments suggest that ventral processing (i.e., vision for perception) dominates size processing in our task, they do not show that the dorsal system was not active at all. Firstly, to show that only the ventral system was active would have required a process-pure task, which cannot be assumed for our task. For example, our task required attentional selection of stimulus and response locations, and the dorsal visual system overlaps with a fronto-parietal system for controlling visual attention (e.g., Corbetta et al., 2009; Marrett et al., 2011). Secondly, there are multiple connections between the two systems, enabling crosstalk between them (cf. Milner, 2017, for a review). We therefore assume that both systems contributed to stimulus processing (and response execution) in our task, but the ventral system with its relative metric dominated the processing of stimulus size.

### Possible accounts of the results

There are two possible accounts for the existence of a correspondence effect between physical stimulus size and left/right responses, ATOM and polarity correspondence. Both accounts explain the existence of this correspondence effect, but only the latter provides a processing model of how correspondence and non-correspondence between stimuli and responses affects performance. ATOM claims the existence of a generalized magnitude processing system, which uses a common metric for processing information about space, time and different kinds of magnitudes that are required for planning and controlling action (Walsh, 2003, 2015). Hence, ATOM predicts the mere existence of a correspondence effect between physical stimulus size and left/right responses, but it neither explains the direction of the effect, nor does it provide a processing model of how the difference between corresponding and noncorresponding conditions arises. Moreover, ATOM is mute as to whether absolute or relative stimulus (size) codes are involved in different kinds of correspondence effects.

According to the polarity-correspondence principle, observers are assigning polarities to pairs of stimuli or responses in classification tasks, if stimuli or responses vary on bipolar dimensions (Proctor & Cho, 2006; Proctor & Xiong, 2015). In particular, several authors suggested that negative polarity is assigned to “small” and “left,” whereas positive polarity is assigned to “large” and “right” (e.g., Lakens, 2012; Proctor & Cho, 2006; Wühr & Heuer, 2021). As a result, and consistent with the assumptions of dual-route models, correspondence of S-R polarities (e.g., small-left, large-right) facilitates response selection, whereas non-correspondence of S-R polarities (e.g., small-right, large-left) impedes response selection during the classification task. These effects of polarity correspondence may occur regardless of whether the critical stimulus value is relevant or not.

Most importantly for the current discussion, the polarity correspondence principle is consistent with the fact that relative, and not absolute, stimulus values are responsible for the correspondence effect between physical stimulus size and horizontal response location. Polarity is a relative, and not an absolute, feature in a set of stimuli or responses. As a result, a particular *S* will be tagged as “large” (positive polarity) if it is the largest stimulus in the current set of stimuli, whereas the same *S* will be tagged as “small” (negative polarity) if it is the smallest stimulus in the current set, consistent with our findings. Although Proctor and colleagues proposed the polarity-correspondence principle as an account of S-R compatibility or correspondence effects in binary choice tasks including two stimuli and two responses, the principle can easily be extended to tasks involving more than two stimuli, as long as the stimuli are varying on a bipolar dimension such as size (e.g., Lakens, 2012). In particular, in our experiments, each stimulus set included three stimuli of increasing size. It seems reasonable to assume that, in this situation, the smallest stimulus represents the lower, negative pole, whereas the largest stimulus represents the upper, positive pole of the range spanned by the stimulus set. In this case, the intermediate stimulus would not receive polarity, and therefore no correspondence effect is observed for this stimulus, which is also consistent with our findings.

### Comparison to other compatibility/correspondence effects

The finding that the correspondence effect between physical stimulus size and response location rests on relative stimulus size coincides with previous findings demonstrating that relative numerical size, and not absolute numerical size, interacts with response location in the SNARC effect (e.g., Dehaene et al., 1993; Fias et al., 1996). This similarity of the two correspondence effects seems consistent both with ATOM and with the polarity-correspondence principle, and therefore does not help differentiating between these accounts.

The fact that S-R correspondence effects involving physical stimulus size and numerical stimulus size both arise from relative stimulus size, seems consistent with ATOM because this model proposes a common metric for the processing of different magnitudes (e.g., Walsh, 2003, 2015). ATOM predicts that this metric has a prelinguistic basis and that magnitude processing is further developed within childhood through interactions with the environment. When grasping objects, for example, the processing of several continuous quantities such as physical size, space, time, and speed coincide and are relative in nature. The processing of discrete quantities such as numbers, which we later learn about, is in turn thought to emerge from our ability to process continuous quantities. Walsh (2015) assumes that “the neuronal scaling mechanisms used for [continuous] dimensions with action-relevant magnitude information will be co-opted in development for the scaling of number” (p. 557), implying that the processing of numerical magnitude might also adopt the prelinguistically relative nature of physical magnitude.

The fact that S-R correspondence effects involving physical stimulus size and numerical stimulus size both arise from relative stimulus size, also seems consistent with polarity correspondence because this is an inherently relative feature of stimuli and responses (e.g., Lakens, 2012; Proctor & Cho, 2006). As stimuli can only be marked as small in relation to stimuli being large, the categorization of each dimension has to occur within the dimension’s given range. Polarity is thus assigned to opposing poles existing in relation to each other. Given that stimuli are coded on multiple dimensions as negative and positive polarity, both physical and numerical dimensions should be encoded as relatively small or relatively large within a given stimulus set and thus lead to S-R correspondence effects (Proctor & Cho, 2006; Proctor & Xiong, 2015).

### Limitations and unpredicted findings

The physical size of two-dimensional stimuli may be measured via diameter or area. If stimulus size increases by a constant amount of diameter, as in our experiments, the changes of area increase when stimulus size increases. In fact, in our experiments, the changes of area increased by 200 mm^2^ from one stimulus size to the next. If, however, stimulus size increases by a constant amount of area, the changes of diameter decreases when stimulus size increases. For the present experiments, we deliberately opted for manipulating the size of objects by diameter because diameter, and not area, is a critical variable for shaping the hand when grasping an object (e.g., Jeannerod, 1986; Jeannerod et al., 1995). We cannot exclude different results would be obtained when we had manipulated the size of objects by area, although we do not see an obvious reason for why this should happen.

Another issue is that we have investigated a rather narrow range of stimulus-size values in our experiments. In particular, in our experiments, stimulus size varied between 5 and 60 mm. Again, we have deliberately chosen this range of stimulus sizes because objects with a size in this range can be grasped with one hand by humans, and we presumed that the correspondence effect between physical stimulus size and left/right responses reflects an adaptation of the motor system to grasping objects of different size. On the other hand, however, we continuously perceive and interact with objects of much larger sizes in everyday life, and it might be an interesting question for future research as to whether the results of the present study generalize to larger objects.

In Experiment 1 we obtained an unpredicted and bothersome main effect of stimulus size on the RTs in the color-discrimination task, where participants responded faster to larger than to smaller objects (see, Richter & Wühr, 2022, for a similar finding). We explained this effect by assuming that the difficulty of color discrimination may depend on stimulus size, and this assumption is confirmed by studies showing that color-discrimination improves when stimulus size increases (e.g., Brown, 1952; Nagy, 1994). The potential problem here is that differences in task difficulty with different stimulus sizes may affect (the size of) the S-R correspondence effect, and thus compromise the objective of our study. Therefore, we tried to avoid this problem in Experiment 2 by changing the relevant stimulus dimension from color to shape, and this change successfully eliminated the main effect of stimulus size in the shape-discrimination task, but left the remaining pattern of results unchanged.

### Conclusion

The results of our study suggest that participants are inadvertently classifying stimuli according to their size in a context-specific manner, that is, with regard to the range of stimulus sizes in the current task. The resulting stimulus-size tags then activate pre-existing associations between “small” and “left” and between “large” and “right,” respectively, producing a correspondence effect between physical stimulus size and left/right responses.

## Data Availability

The datasets of Experiment 1 and 2 have been published on the “SowiDataNet|datorium“ repository (10.7802/2391).

## Footnotes

1 We use the term “compatibility effect” to describe the effect of different mappings between relevant stimulus and relevant response features. In contrast, we use the term “correspondence effect” to describe the effect of different combinations between irrelevant stimulus features and relevant response features.

2 The physical size of an object may be measured by size (width) or by area. In fact, increasing the size of visual stimuli by a constant diameter (width) produces increasing changes of area, whereas increasing stimulus size by area would produce decreasing changes of diameter. For our experiments, we deliberately decided to increase the size of the stimuli by a constant diameter (width) because diameter, and not area, is a critical parameter for grasping objects (e.g., Jeannerod, 1997; Jeannerod et al., 1995).

3 A similar observation is reported in Richter and Wühr (2022).
